# Contribution of potassium channels, beta2-adrenergic and histamine H1 receptors in the relaxant effect of baicalein on rat tracheal smooth muscle

**DOI:** 10.22038/ijbms.2019.36377.8666

**Published:** 2019-11

**Authors:** Saeideh Saadat, Javad Boskabadi, Mohammad Hossein Boskabady

**Affiliations:** 1 Neurogenic Inflammation Research Center, Mashhad University of Medical Sciences, Mashhad, Iran; 2 Department of Physiology, Faculty of Medicine, Mashhad University of Medical Sciences, Mashhad, Iran

**Keywords:** Baicalein, Beta2-adrenergic, Histamine H1 receptors, Relaxation, Smooth muscle, Trachea

## Abstract

**Objective(s):**

Baicalein, a compound extracted from a variety of herbs, showed various pharmacological effects. This study evaluated the relaxant effects of baicalein and its underlying molecular mechanisms of action on rat’s isolated tracheal smooth muscle.

**Materials and Methods:**

Tracheal smooth muscle were contracted by 10 μM methacholine or 60 mM KCl and the effects of cumulative concentrations of baicalein (5, 10, 20 and 40 mg/ml) and theophylline (0.2, 0.4, 0.6 and 0.8 mM) were evaluated. To examine the possible mechanism(s) of the relaxant effect of baicalein, its effect was also evaluated on incubated tissues with atropine, indomethacin, diltiazem, N(G)-Nitro-L-arginine methyl ester (L-NAME), glibenclamide, propranolol and chlorpheniramine.

**Results:**

A concentration-dependent and significant relaxant effect was seen for baicalein in non-incubated tissues contracted by KCl or methacholine (*P<*0.01 to *P<*0.001). No significant difference was seen between the relaxant effects of high concentrations of baicalein and theophylline. The relaxant effects of all concentrations of baicalein in incubated tissues with glibenclamide, propranolol and chlorpheniramine were significantly lower than non-incubated tissues (*P<*0.05 to *P<*0.001). Additionally, the EC_50_ values of baicalein in incubated tissue with propranolol was significantly higher than non-incubated condition (*P<*0.05).

**Conclusion:**

A potent relaxant effect comparable to the effect of theophylline was shown for baicalein, which was probably mediated via inhibition of histamine (H1) receptors, stimulation of beta2-adrenergic receptors and potassium channels activation.

## Introduction

Flavonoids comprise a large group of naturally existing polyphenolic compounds widely distributed throughout the plant kingdom (1). The flavonoids, baicalin and its aglycone, baicalein (5,6,7-trihydroxy-2-phenyl-4H-1-benzopyran-4-one) are found in edible medicinal plants, *Scutellaria baicalensis *Georgi*, Scutellaria viscidula *Bge*, Scutellaria likiangensis *Diels*, Scutellaria amoenac *H. Wright*, Scutellaria rehderiana Diels, Scutellaria hypericifolialevl, Oroxylum indicum *L. Kurz and* Plantago major* in abundant quantities (2-6). The anti-inflammatory and antioxidant effects of these flavonoids were demonstrated in various disease models (3, 7, 8). Flavonoids also modulate vascular tone and the potency of relaxant effect of flavonoids were reported as follow; flavonols > flavones > flavanols (1).

Various neurotransmitters, mediators and drugs influence airway smooth muscle (ASM) which most of them are mediated by cell surface receptors. Therefore, various bronchodilators and bronchoconstrictors have now been identified (9). Although, the vasodilatory effects of baicalein have been reported (10) but so far, the relaxant effect of baicalein has not been shown on the ASM. Within such a context, the aim of the present study was evaluation of the relaxant effects of baicalein and its underlying molecular mechanisms of action in rat’s isolated tracheal smooth muscle (TSM).

## Materials and Methods

***Materials***

Baicalein (C_15_H_10_O_5_) with CAS Number 491-67-8 was purchased from Sigma Chemical Co Ltd. Potassium chloride (KCl) was obtained from Merck (Darmstadt, Germany). Methacholine, atropine, chlorpheniramine, indomethacin, diltiazem, glibenclamide, propranolol, and N(G)-Nitro-L-arginine methyl ester (L-NAME) were also purchased from Sigma Chemical Co, Ltd.

***Animals***

Fifty-six young male Wistar rats (200–250 g) purchased from the Animal House, Faculty of Medicine, Mashhad University of Medical Sciences (Mashhad, Iran). The animals were maintained under controlled condition at 12/12 hr light/dark cycle and 22 ± 2 ^°^C. Water and food *ad libitum *was always accessible to animals. The Ethics Committee of Mashhad University of Medical Sciences (Code; 941083) confirmed the study protocol. The study was carried out according the regulations of the Institute of Laboratory Animals Resources Commission on Life Sciences (11).

***Preparation of tracheal ring***

Tracheal rings of rats were prepared, mounted in 10 ml organ bath containing Krebs-Henseliet solution (KHs), and maintained at 37 ± 0.5 ^°^C with isometric tension of 1 g as previously described (12, 13). In all experiments, contraction responses were measured using an isometric transducer (MLT0202, AD Instruments, Australia) which was connected to a power lab system (Power Lab 8/30, ML870, AD Instruments, Australia). 

***Measurement of tracheal smooth muscle relaxation***

**Figure 1:**
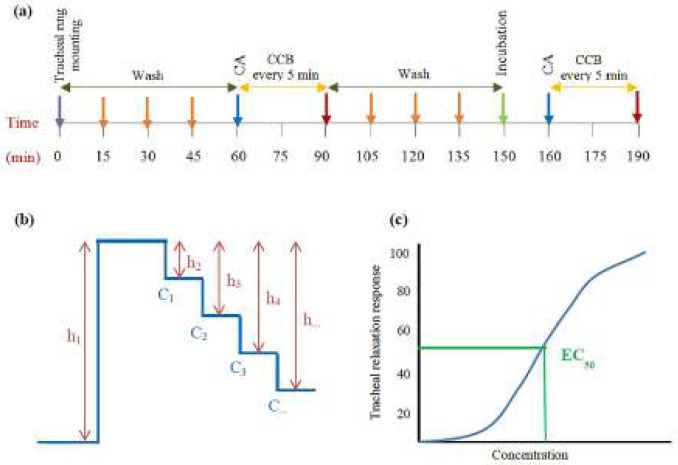
(a) Time course of the examining of the relaxant effect, (b) Maximum contraction due to contractile agent (CA, ie 10 µM methacholine or 60 mM KCl, h1) and concentration-response relaxant effect due to cumulative concentration (C) of baicalein (CCB), (h2, h3, h4 and h...), and (c) concentration-response curve of the relaxation effect of baicalein and the method of measurement of EC_50_. Each tissue was equilibrated for at least 1 hr, while it was washed with Krebs-Henseliet solution every 15 min before examining the relaxant effect

TSM relaxation was examined according to the method described previously (12, 13). Briefly, TSM was contracted by 10 μM methacholine for 7 min or 60 mM KCl for 5 min and the cumulative concentrations of baicalein (5, 10, 20 and 40 mg/ml) (14), theophylline (0.2, 0.4, 0.6 and 0.8 mM) as a positive control, or 1 ml of normal saline (NS) as a negative control were added to the tissue bath every 5 min (Figure1).

The concentration-response curves of the relaxant effect of baicalein was constructed in each experiment and its effective concentration causing 50% of maximum response (EC_50_) was calculated as previously described (12). 

***Experimental groups***

In order to examine the possible mechanism(s) of the relaxant effect of baicalein (15), its relaxant effect was evaluated in various groups as described in Table 1.

***Statistical analysis***

The results were described as the mean±SEM. The comparison of the results was done using One-way analysis of variance (ANOVA) followed by Tukey’s multiple comparison test. Statistical significance was considered at *P*<0.05.

## Results

***The relaxant effects of baicalein on tracheal smooth muscle contracted by KCl***

**Figure 2:**
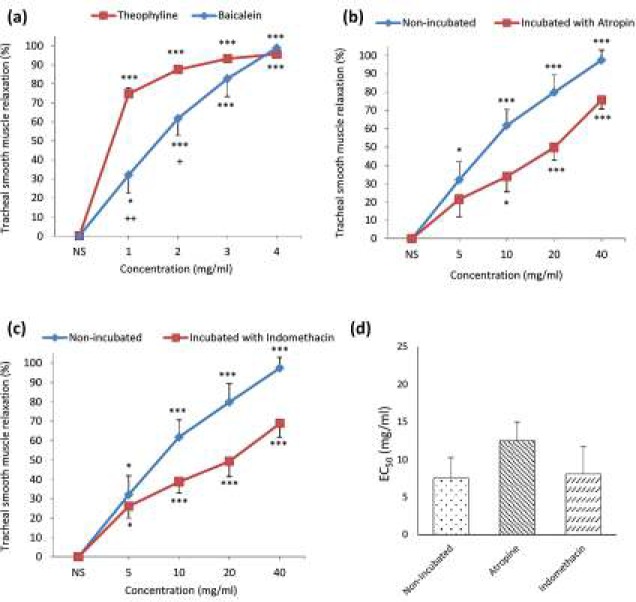
Concentration-response curves of the relaxant effect (mean±SEM) of baicalein and theophylline on KCl (60 mM) induced contraction of tracheal smooth muscle in (a) non-incubated tissues. 1, 2, 3 and 4 in X-axis represent four concentration of baicalein (5, 10, 20 and 40 mg/ml) and theophylline (0.2, 0.4, 0.6 and 0.8 mM). Concentration-response curves of the relaxant effect (mean±SEM) of baicalein on KCl (60 mM) induced contraction of TSM in non-incubated and incubated tissues with (b) atropine (1 μM, n=5) and (c) indomethacin (1 μM, n=7). (d) EC_50_ values of baicalein induced relaxation obtained on contracted TSM of rat with 60 mM KCl in non-incubated (n=7) and incubated tissues with atropine (n=5) and indomethacin (n=7). TSM relaxation was presented as percent change in proportion to maximum contraction due to 60 mM KCl. *: *P<*0.05, ***: *P<*0.001 compared to saline (NS), +: *P<*0.05, ++: *P<*0.01 compared to the effect of theophylline

Concentration-dependent and significant relaxant effect was seen for baicalein in the tissues contracted by KCl (*P*<0.05 for 5 mg/ml and *P*<0.001 for 10, 20 and 40 mg/ml). The relaxant effects of 5 and 10 mg/ml of baicalein were significantly lower than theophylline (*P*<0.05 and *P*<0.01, respectively, Figure 2a).

Baicalein showed concentration-dependent and significant relaxant effects in incubated TSM with atropine (*P*<0.05 for 10 mg/ml and *P*<0.001 for 20 and 40 mg/ml, Figure 2b) and indomethacin (*P*<0.05 for 5 mg/ml and *P*<0.001 for 10, 20 and 40 mg/ml, Figure 2c). No significant difference was observed in the relaxant effects of baicalein between non-incubated and incubated tissue with atropine or indomethacin (Figure 2).

There was no significant difference in EC_50_ values of baicalein between non-incubated tissues concentrated by KCl (7.5±2.73) and incubated with atropine (12.5±2.49) or indomethacin (8.1±3.58, Figure 2d).

***The relaxant effects of baicalein on tracheal smooth muscle contracted by methacholine***

**Figure 3:**
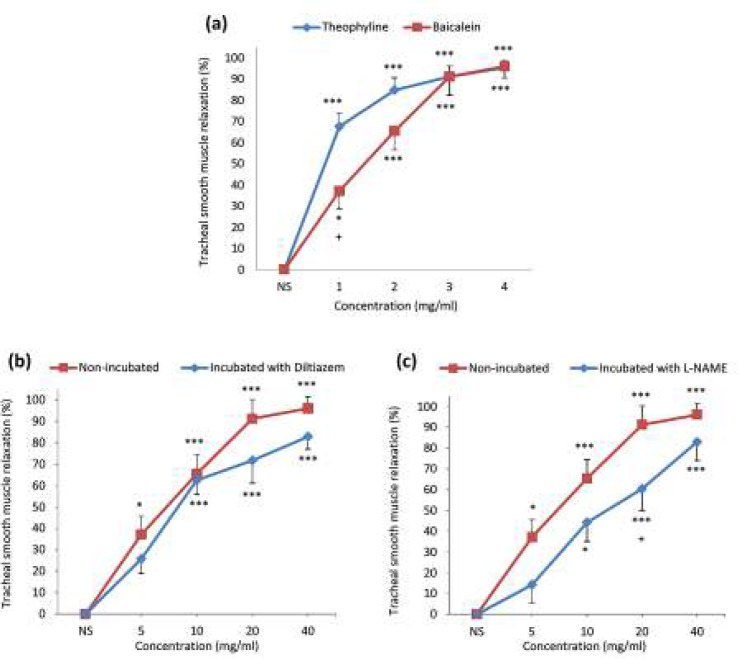
Concentration-response curves of the relaxant effect (mean±SEM) of baicalein and theophylline on methacholine (10 μM) induced contraction of tracheal smooth muscle in (a) non-incubated tissues. 1, 2, 3 and 4 in X-axis represent four concentrations of baicalein (5, 10, 20 and 40 mg/ml) and theophylline (0.2, 0.4, 0.6 and 0.8 mM). Concentration-response curves of the relaxant effect (mean±SEM) of baicalein on methacholine (10 μM) induced contraction of TSM in non-incubated (n=7) and incubated tissues with (b) diltiazem (5 μM, n=6) and (c) L-NAME (300 μM, n=6). TSM relaxation was presented as percent change in proportion to maximum contraction due to 10 μM methacholine. *: *P<*0.05, ***: *P<*0.001 compared to saline (NS). +: *P<*0.05 compared to the effect of theophylline

Concentration-dependent and significant relaxant effect was observed for baicalein in the tissues contracted by methacholine (*P*<0.05 for 5 mg/ml and *P*<0.001 for 10, 20 and 40 mg/ml, Figure 3a). There was no significant difference between the relaxant effects of 10, 20 and 40 mg/ml of baicalein and theophylline, but the relaxant effect of 5 mg/ml of baicalein was significantly lower than theophylline (*P*<0.05, Figure 3a).

In incubated TSM with diltiazem, baicalein showed a concentration-dependent and significant relaxant effect (*P*<0.001 for 10, 20 and 40 mg/ml). There was no significant difference between the relaxant effects of baicalein in non-incubated and incubated tissues with diltiazem (Figure 3b).

Concentration-dependent and significant relaxant effect were seen for baicalein in incubated tissues with L-NAME (*P*<0.05 for 10 mg/ml and *P*<0.001 for 20 and 40 mg/ml, Figure 3c). The relaxant effects of 20 mg/ml of baicalein in incubated tissues with L-NAME was significantly lower than non-incubated TSM (*P*<0.05 Figure 3c).

**Figure 4:**
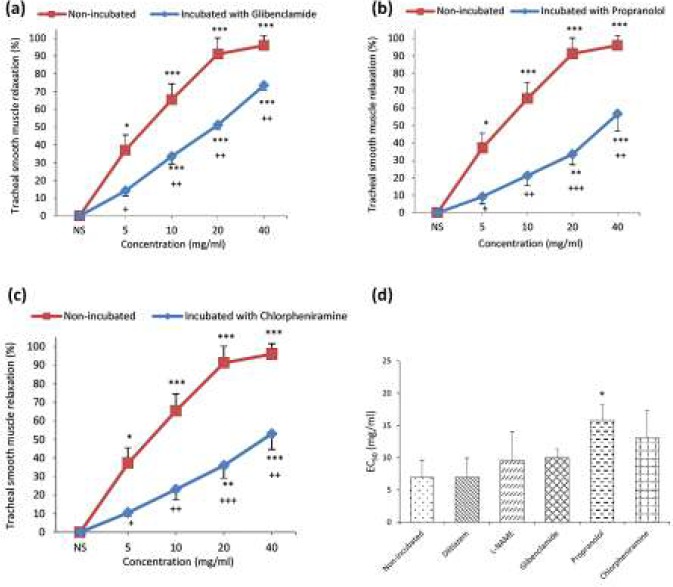
Concentration-response curves of the relaxant effect (mean±SEM) of baicalein on methacholine (10 μM) induced contraction of tracheal smooth muscle in non-incubated (n=6) and incubated tissues with (a) glibenclamide (1 μM, n=7), (b) propranolol (1 μM, n=6), (c) chlorpheniramine (1 μM, n=6). (d) EC_50_ values of baicalein induced relaxation obtained on contracted TSM of rat with methacholine (10 μM) in non-incubated (n=6) and incubated tissues with diltiazem (n=6), L-NAME (n=6), glibenclamide (n=7), propranolol (n=6), and chlorpheniramine (n=6). TSM relaxation was presented as percent change in proportion to maximum contraction due to 10 μM methacholine. *: P<0.05, **: *P<*0.01, ***: *P<*0.001 compared to saline (NS), and +: *P<*0.05, ++: *P<*0.01, +++: *P<*0.001 compared to non-incubated tissues

In incubated TSM with glibenclamide, a concentration-dependent and significant relaxant effect was seen for baicalein (*P*<0.001 for 10, 20 and 40 mg/ml, Figure 4a). The relaxant effects of all concentrations of baicalein in incubated tissues with glibenclamide were significantly lower than non-incubated TSM (*P*<0.05 for 5 mg/ml and *P*<0.01 for 10, 20 and 40 mg/ml, Figure 4a).

 Only two last concentrations of baicalein showed significant relaxant effects in incubated tissues with propranolol (*P*<0.01 and *P*<0.001 for 20 and 40 mg/ml, respectively, Figure 4b) and incubated tissues with chlorpheniramine (*P*<0.01 and *P*<0.001 for 20 and 40 mg/ml, respectively, Figure 4c). The relaxant effects of all concentrations of baicalein in incubated tissues with propranolol and chlorpheniramine were significantly lower than non-incubated TSM (*P*<0.05 to *P*<0.001, Figure 4b and 4c).

No significant difference was observed in EC_50_ values of baicalein between non-incubated and contracted tissues by methacholine (6.9±2.75) and incubated tissue with chlorpheniramine (13.1±4.295), diltiazem (7.0±2.93), glibenclamide (10±1.355) or L-NAME (9.5±4.51). The EC_50_ values of baicalein in incubated tissue with propranolol (15.8±2.47) was significantly higher than non-incubated condition (*P*<0.05, Figure 4d).

Relaxation response due to first concentration = h2 / h1×100

Relaxation response due to second concentrations = h3 / h1×100

Relaxation response due to third concentrations = h4 / h1×100

Relaxation response due to … concentrations = h… / h1×100

**Table 1:** The protocol of the study and the methods of evaluating of various mechanisms of the relaxant of effect of baicalein on tracheal smooth muscle

Contracture agent	Condition	Incubating substance	Mechanisms
60 mM KCl	Non-incubated tissues (n=7)		
	Incubated tissues	1 μM atropine (n=5)	Muscarinic receptor inhibition
		1 μM indomethacin (n=7)	Cyclooxygenase inhibition
10 μM methacholine	Non-incubated tissues (n=6)		
	Incubated tissues	1 μM chlorpheniramine (n=6)	Histamine (H1) receptor inhibition
		5 μM diltiazem (n=6)	Calcium channel blocking
		1 μM glibenclamide (n=7)	Potassium channel opening
		1 μM propranolol (n=6)	β-adrenoceptor stimulation
		300 μM L-NAME (n=6)	Nitrite oxide (NO) production

L-NAME: N(G)-Nitro-L-arginine methyl ester

**Figure 5:**
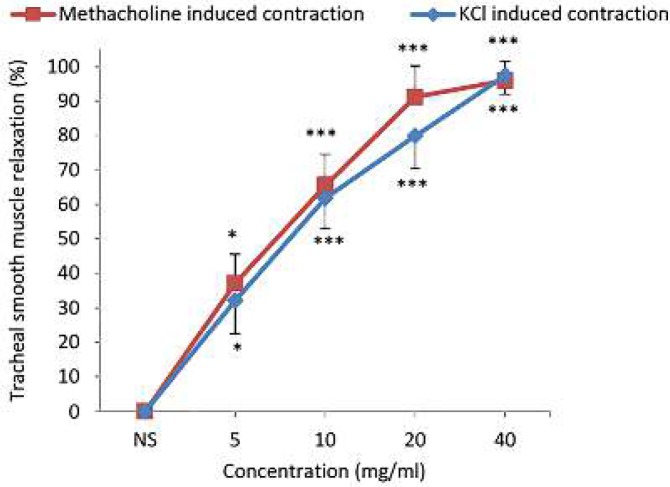
Concentration-response curves of the relaxant effect (mean±SEM) of baicalein on methacholine (10 μM, n=6) and KCl (60 mM, n=7) induced contraction of non-incubated tracheal smooth muscle. The relaxation response was presented as percent change in proportion to maximum contraction response due to 10 μM methacholine or 60 mM KCl. *: *P<*0.05, ***: *P<*0.001 compared to saline (NS)

**Table 2:** Comparison of the relaxant effect of baicalein (percentage change in proportion to the maximum contraction) in different incubated TSM contracted by 10 μM methacholine or 60 mM KCl

Concentration (mg/ml)				Incubating substance	Contracting substance
40	20	10	5		
**75.62±4.98**	49.75±7.06	33.88±8.43	21.53±9.55	Atropine	KCl
**68.82** **±7.16**	49.33±8.10	38.72±5.73	26.11±6.06	Indomethacin	
**82.90±5.86 ** ^*+^	71.75±10.73 ^*+^	62.53±6.69^***++^	25.78±6.90	Diltiazem	Methacholine
**83.00±9.02 ** ^+^	60.41±10.56	44.25±9.21	14.23±8.83	L-NAME	
**73.62±2.71 ** ^+^	51.27±2.37 ^*^	33.56±4.39	14.24±2.95	Glibenclamide	
**56.76±9.88**	33.63±6.05	21.40±5.71	9.29±4.21	Propranolol	
**53.08±8.59**	35.98±6.90	23.15±5.71	10.61±2.02	Chlorpheniramine	

Data were presented as mean±SEM. *: *P<*0.05, ***: *P<*0.001 compared to incubated tissues with propranolol. +: *P<*0.05, ++: *P<*0.01 compared to incubated tissues chlorpheniramine

TSM: tracheal smooth muscle; L-NAME: N(G)-Nitro-L-arginine methyl ester

***Comparison of the relaxant effects of baicalein in tissues contracted by KCl with those of methacholine***

There was no significant difference in the relaxant effects of various concentrations of baicalein obtained in KCl-induced contraction with those in TSM contracted by methacholine (Figure 5).

## Discussion

The results of this study showed concentration-dependent relaxant effects of baicalein in non-incubated TSM contracted by KCl or methacholine, which may indicate possible bronchdilatory effect of this agent in airway of patient with airway constriction.

In TSM incubated with atropine, indomethacin, diltiazem and L-NAME, there were no significant difference in the relaxant effects of baicalein between non-incubated and incubated tissues. These results indicated that the relaxant effect of baicalein is not due to muscarinic receptors, arachidonic acid metabolism and cyclooxygenase pathways, calcium channel blocking and NO production. 

To evaluate the effect of baicalein on histamine (H1) receptors, beta2-adrenergic receptors and potassium channel activation, the relaxant effects of baicalein were examined on TSM incubated with chlorpheniramine, propranolol and glibenclamide, respectively. The relaxant effects of all concentrations of baicalein in incubated tissues with chlorpheniramine, propranolol and glibenclamide were significantly lower than non-incubated tissues. These results indicated inhibitory effect of baicalein on histamine (H1) receptors, its stimulatory effect on beta2-adrenergic receptors and its opening effect on potassium channels are responsible in its relaxant effects on TSM. Lower relaxant effect of some concentrations of baicalein on incubated tissues with chlorpheniramine and propranolol compared to the effects obtained in tissues incubated with other agent also support this mechanism of action for baicalein (Table 2). In addition, EC_50_ baicalein in incubated tissues with propranolol was significantly higher than that of non-incubated TSM, which supports the stimulatory effect of this agent on beta2-adrenergic receptors. Taken together, these findings suggest the possible inhibitory effect of baicalein on histamine (H1) receptors, its stimulatory effect on beta2-adrenergic receptors and its activation effect on potassium channels.

Baicalein have been thought to be as the inhibitory agent for chemical mediator release from mast cells *in vitro* and allergic immediate phase reactions in skin and airway *in vivo *(16). The bronchoconstrictory effect of histamine is mediated via H1 receptors (9). Until now, the relaxant effect of baicalein on TSM has not been reported, while baicalin showed anti-asthmatic activity in isolated tracheal muscle from asthmatic guinea pigs (17).

Biological activity studies have indicated that baicalein has a beta1-adrenergic receptors antagonistic effect (6), while the relationship between baicalein and beta2-adrenergic receptors is unknown. Probably, baicalein stimulates beta2-receptors and increases production of cyclic adenosine monophosphate (cAMP), which leads to the characteristic cellular response via the activation of protein kinase A (PKA). In ASM cells, PKA phosphorylates acertain potassium channel opener, leads to potassium efflux from the cell, membrane hyperpolarization, and relaxation.

The predominant K1 channel in ASM is the maxi-K channel, which may be opened by cAMP, but also through direct coupling of beta2-receptors via Gs proteins (9). Thus, beta2-agonists may cause bronchodilatation via a direct effect of maxi-K channels as well as through an increase in cAMP (9). Glibenclamide is known to block ATP-dependent potassium channels. The present study suggests that baicalein probably activates ATP-dependent potassium channels, while activation of glibenclamide-sensitive potassium channels was not involved in baicalein-induced relaxation in endothelium-denuded arteries (14). Therefore, bacalein could induce bronchodilation by maxi-K channel opening.

In other smooth muscles, baicalein inhibited lipoxygenase, resulting in reduced biosynthesis and release of arachidonic acid derived vasoconstrictor products such as aortic smooth muscle cells (18). Baicalein relaxed the arterial smooth muscle partially at higher concentrations through inhibition of the contractile mechanisms mediated by protein kinase C (14). On the other hand, baicalein increased vasoconstricting sensitivity to adrenergic agonist in isolated rat arteries (18, 19). It is suggested that baicalein induces a contractile response at low concentrations and inhibits the endothelium-dependent relaxation, probably through inhibition of endothelial NO formation or release (14). This flavonoid impaired the endothelium independent relaxation by NO donors and attenuates NO-mediated aortic relaxation and cyclic GMP increases, likely through inhibition of NO-dependent guanylate cyclase activity (18). Baicalein reduced both acetylcholine and cyclopiazonic acid induced relaxation. It may also has little influence on the nifedipine-sensitive calcium channels or caffeine-sensitive intracellular calcium release in arterial smooth muscle cells (14). However, the current study did not show the effect of baicalein on calcium channel blocking, NO formation, arachidonic acid metabolism and cyclooxygenase pathways.

## Conclusion

The present study provides novel information about the tracheal smooth muscle relaxant effect of baicalein. The relaxant effect of baicalein on TSM probably mediated through inhibition of histamine (H_1_) receptors, stimulation of beta2-adrenergic receptors and potassium channels activation.
